# Recent Advances in the Detection of Indoor Fungi

**DOI:** 10.3390/pathogens12091136

**Published:** 2023-09-06

**Authors:** Donát Magyar

**Affiliations:** National Center for Public Health and Pharmacy, 1097 Budapest, Hungary; magyar.donat@gmail.com

According to reviews carried out by numerous studies from different geographic areas and by several scientific bodies, including the WHO [1], rising damp or mould has consistently been shown to be associated with a variety of adverse health effects. The most notable diseases that moulds can cause are allergies, hypersensitivity pneumonitis, and infection. The prevalence of mould allergy is approximately 5% to 30% of atopic patients [2].

Indoor mould is an emerging problem due to recent trends in the construction industry. Once wet, modern building structures (e.g., those with drywall, dropped ceiling, and fibrous insulation materials), offer a favourable environment for fungal growth, which often remains unnoticed by residents [3,4,5].

Conventional mould detection methods, such as the visual observation of colonies and air sampling, are often not sufficient to detect hidden mould in modern buildings, where an innovative approach is needed.

The composition of fungal species and their (toxic) metabolites is changing due to the new environmental conditions offered by new building materials with different physical and chemical properties. Therefore, it is important to remain vigilant regarding the constantly changing microbial environment of our buildings. This Special Issue in *Pathogens* aims to contribute to the knowledge of indoor detection methods, the spectra of fungal species present in buildings, their metabolites, and their health effects.

The advantages of certain sampling methods were presented in two papers in our Special Issue [6,7]. Viegas et al. [6] reviewed sixteen studies conducted in Portugal between 2018 and 2021 that used electrostatic dust cloths (EDC) to evaluate fungal exposure. The findings suggest that this passive sampling method has many advantages. For example, it can be used in sampling campaigns to detect a wide range of fungi and their metabolites, and its simple extraction protocol obtains a liquid sample and facilitates the application of various assays. In addition, EDC is low-cost and causes minimal disruption to residents’ (or workers’) daily routines.

The contribution of surface fungi to airborne fungal concentrations was evaluated with air and surface sampling combined with digital image quantification of surface mould spots in households in Taiwan [7]. To quantify the sampling area, the authors calculated the target surface pixels in digital images in Photoshop. The airborne concentration showed no significant correlation with the fungal concentrations of most fungi on surfaces, except *Geotrichum*. On the other hand, the surface area of visible mould spots was significantly related to the airborne concentration of some fungi, including Aspergillus and *Geotrichum*. The authors recommend the quantification of the surface fungal area to assess the risk represented by visible moulds.

In the field of new detection methods, Chauhan et al. [8] implemented next-generation sequencing approaches to explore the bacterial and fungal communities and their abundance in dust samples collected from air vents and ground surfaces. Regardless of the visibility of fungi in the homes, the abundance of potentially pathogenic fungal species was similar. The authors pointed out that molecular methods provide a wealth of information about fungi and bacteria, which is very useful for both diagnostics and risk assessment.

In another study on the fungal composition of indoor dust, Andersson et al. [9] focused on the cultivable microbiota in sport facilities subjected to intense cleaning and disinfection during the COVID-19 pandemic. Strains of *Aspergillus flavus* and *A. fumigatus* classified as risk group 2 (RG-2) and bio-safety level 2 (BSL-2) were detected in the samples. Low viable counts of bacteria and a high proportion of potentially pathogenic and stress-tolerant fungi were found, which may indicate certain unexpected and unwanted effects of the intense disinfection. These measures have apparently eliminated most viable microorganisms, except for potentially pathogenic and stress-tolerant fungi.

Indoor dust was used to investigate the association between teachers’ individual work-related symptoms and the intrinsic in vitro toxicity in classrooms by Salin et al. [10]. The toxicity of wiped dust was used to measure the mitochondrial dysfunction with boar sperm motility inhibition assay. The toxicity of cultured microbes was found to be associated with nine symptoms, including eye discharge, increased need for sleep, and sore throat.

Paavanen-Huhtala et al. [11] introduced an easy, comprehensive, and ethically acceptable in vivo model system to monitor the response of multicellular organisms to indoor air toxicity using transgenic *Caenorhabditis elegans* nematode strains carrying stress-responsive fluorescent reporters. The authors evaluated the nematodes’ ability to sense toxins, especially those that are present in fungi or in moisture-damaged building materials. The tests reproducibly demonstrated time- and dose-dependent fluorescent responses in liquid and air samples. The results could be quantitated by either microscopy or spectrometry.

Jakšić et al. [12] presented a study on the cytotoxic, genotoxic, and pro-inflammatory properties of metabolite mixtures obtained from fungi on human adenocarcinoma cells (A549) and monocytic leukemia cells induced in macrophages (THP-1 macrophages). Metabolites of the most common Aspergillus species isolated from indoor air samples were used. The combined toxins showed both additive/synergistic (*Aspergillus* series *Nigri*+*Flavi*) and antagonistic (*Nigri*+*Versicolores*) effects. Combined, these Aspergilli have a more pronounced impact on cytokine excretion by THP-1 macrophages than the same Aspergilli applied separately, suggesting a more significant effect on immunomodulation following exposure to mixtures of fungal metabolites.

The genus *Aspergillus*, represented by well-known pathogenic species, was the most diverse and dominant genus among the 284 airborne fungi collected from buildings [13]. Both “ordinary” and “problematic” buildings contained potentially pathogenic fungi. Screening fungal strains for their ability to grow at 37 °C and 30 °C, and at neutral pH, has been a successful method for detecting potentially pathogenic indoor isolates and monitoring their diversity.

In their study, Micheluz et al. [14] revealed how *Eurotium halophilicum* (a psychrotolerant and halophilic species) manipulates its environment to survive extreme environmental conditions using samples taken from the surfaces of library books. This fungus achieves extreme stress tolerance with eugsterite and mirabilite crystals embedded within extracellular polymeric substances, and with water-absorbing hair-like microfilaments which maintain the conditions consistent with its water-activity/humidity requirements for growth.

A rare genus not previously detected In Finnish buildings, *Acrostalagmus luteoalbus*, was the focus of the study conducted by Andersson et al. [15]. The fungus was found to be the major constituent of the mixed microbiota in the wet cork liner from an outdoor wall. The strain produced the immunosuppressive and cytotoxic melinacidins II, III, and IV, as evidenced by mass spectrometry analysis. Since the airborne concentration of conidia was too low to explain the reported symptoms, the authors hypothesized that the symptoms were triggered by microbial metabolites migrating from the wet outdoor wall to the indoor space.

A new species, *Dichotomopilus finlandicus*, was described from indoor environments by Kedves et al. [16]. The fungus was discovered among *Chaetomium*-like strains isolated from indoor environments in Hungary and Finland, two geographically distant regions of Europe with drier and wetter continental climates, respectively. The growth of the isolates was examined at different temperatures, while their extracellular enzyme production was determined spectrophotometrically. The enzyme production of the strains proved to be diverse and isolate-dependent, having no correlation with either the isolation site or the growing substrate.

Another new species and genus, *Hagnosa longicapillata*, was described and illustrated from indoor environments [17]. To place this fungus in Sordariales, the new family, Hagnosaceae, is proposed. The fungus was isolated from wooden building materials, where it was quite common in Hungary. The ostiolar region of the perithecia is ornamented with a unique five-lobed, flower-shaped crown. The short-distance dispersal of the ascospores is facilitated by the mechanical disturbance of the mycelial web. However, long-distance airborne dispersal is limited due to the relatively large size of the spores, thus they are transported by ants visiting the buildings. The ants harvest and use the mycelia to insulate their nest.

The contributors to this Special Issue provided insights into current issues in indoor mycology, particularly from a methodological [6,7,8,11,13], metabolic/toxicological [10,11,12,14,15], and taxonomical [8,9,13,16,17] point of view. Overall, two key messages emerge: (i) the current detection methods appear to be shifting from air sampling [11,12] to dust sampling [6,8,9,10,13], as the latter provides information on longer exposure periods and a higher biodiversity; (ii) the microbial composition of buildings is still largely unknown [9,13,16,17]. Our authors demonstrated that the indoor environment should also be a focus of taxonomical research, especially with regard to wooden and cellulosic materials [14,15,16,17], which proved to have a relatively high biodiversity. The studies have highlighted that further research efforts are needed to fill the large knowledge gaps on the metabolites of indoor fungi in order to understand their interactions and health effects [12,15].

In recent times, the diversity of new scientific findings indicates that indoor mycology is in a “historical era of continental discovery”. In the coming years, new detection methods and accumulating scientific information will hopefully lead to a clearer understanding of the health effects of indoor fungi, allowing for risk assessment and the development of guidelines to prevent harmful microbial processes in the indoor environment.
